# Genetic factors associated with small for gestational age birth and the use of human growth hormone in treating the disorder

**DOI:** 10.1186/1687-9856-2012-12

**Published:** 2012-05-15

**Authors:** Paul Saenger, Edward Reiter

**Affiliations:** 1 Albert Einstein College of Medicine, Winthrop University Hospital, 120 Mineola Boulevard, Mineola, NY, 13501, USA; 2 Baystate Children’s Hospital, Tufts University School of Medicine, 759 Chestnut Street, Springfield, MA, 01199, USA

**Keywords:** Growth hormone, Small for gestational age, Insulin-like growth factor, Acid-labile subunit deficiency, Uniparental disomy

## Abstract

The term small for gestational age (SGA) refers to infants whose birth weights and/or lengths are at least two standard deviation (SD) units less than the mean for gestational age. This condition affects approximately 3%–10% of newborns. Causes for SGA birth include environmental factors, placental factors such as abnormal uteroplacental blood flow, and inherited genetic mutations. In the past two decades, an enhanced understanding of genetics has identified several potential causes for SGA. These include mutations that affect the growth hormone (GH)/insulin-like growth factor (IGF)-1 axis, including mutations in the IGF-1 gene and acid-labile subunit (ALS) deficiency. In addition, select polymorphisms observed in patients with SGA include those involved in genes associated with obesity, type 2 diabetes, hypertension, ischemic heart disease and deletion of exon 3 growth hormone receptor (d3-GHR) polymorphism. Uniparental disomy (UPD) and imprinting effects may also underlie some of the phenotypes observed in SGA individuals. The variety of genetic mutations associated with SGA births helps explain the diversity of phenotype characteristics, such as impaired motor or mental development, present in individuals with this disorder. Predicting the effectiveness of recombinant human GH (hGH) therapy for each type of mutation remains challenging. Factors affecting response to hGH therapy include the dose and method of hGH administration as well as the age of initiation of hGH therapy. This article reviews the results of these studies and summarizes the success of hGH therapy in treating this difficult and genetically heterogenous disorder.

## Definition and epidemiology of small for gestational age (SGA)

Despite past inconsistencies in defining small for gestational age (SGA) (as reviewed by Saenger et al [1]) the International Societies of Pediatric Endocrinology and the Growth Hormone Research Society, as well as the International Small for Gestational Age Advisory Board, recently recommended that the term refer to infants whose birth weights and/or lengths are at least two standard deviation (SD) units less than the mean for gestational age [2,3]. According to this definition, approximately 3%–10% of newborns are considered SGA at birth, although it should be noted that new intrauterine growth curves created with a more contemporary, larger, and more racially diverse population suggest that many SGA patients are often misclassified as appropriate for gestational age (AGA) [4]. While most of these infants undergo catch-up growth, 10%–15% remain small for their age at the age of 2 years [5-8]. In 2001, human growth hormone (hGH) therapy using dose regimens up to 48 mcg/kg/week [3] was approved by the United States (US) Food and Drug Administration (FDA) to treat SGA patients greater than 2 years old. However, because the causes of SGA are diverse, hGH treatment outcomes vary among patients. Thus, identifying the underlying mechanisms for SGA births may help predict patient response to hGH therapy. Causes for SGA births, which are summarized in Table 1[3,4], involve environmental factors, placental factors such as abnormal uteroplacental blood flow, or inherited genetic mutations [3,4]. Over the last two decades, significant research related to genetic mutations that influence SGA has been conducted, and this article reviews the results of these studies and summarizes the success of hGH therapy in treating this condition. It should be mentioned at the beginning of this review, however, that the number of genetic variations for any particular gene that has been associated with SGA birth does not necessarily correlate with the number of patients who have this defect. For instance, four different genetic mutations in the distal region of the terminal long arm of chromosome 15 linked with SGA birth size will be described, while only two mutations are illustrated for patients born SGA with Silver-Russell syndrome (SRS). However, this does not mean that SGA patients are two times more likely to have a mutation in the distal region of chromosome 15 than a mutation associated with SRS. Of note, a website from the Growth Genetics Consortium, an international collaboration, gathers all current information about genetic syndromes disrupting the growth hormone and insulin-like growth factor (IGF) axis [9]. Cases reported involve the following genes: GHR, GHRHR, STAT5B, IGF1, IGF2, IGFALS, and IGF1R. Forty-eight cases have been approved for inclusion into the database so far. This paper aims to illustrate the variety of genetic mutations that are associated with SGA births while concurrently describing how other phenotype characteristics of the patient, such as motor or mental development, can vary depending on which mutation was inherited. 

**Table 1:** Factors associated with increased incidence of SGA birth

**Fetal**	**Maternal**	**Uterine/Placental**	**Demographic**
**Karyotypic**	**Medical conditions**	**Gross structural placental factors**	**Maternal age**
-Trisomy 21	-Hypertension	-Single umbilical artery	-Very young age
-Trisomy 18	-Renal disease	-Placental hemangiomas	-Older age
-Monosomy X	-Diabetes mellitus	-Infarcts, focal lesions	**Maternal height**
-Trisomy 13	-Collagen vascular diseases	**Insufficient uteroplacental perfusion**	**Maternal weight**
**Chromosomal abnormalities**	-Maternal hypoxemia	-Suboptimal implantation site	**Maternal and paternal race**
-Autosomal deletion	**Infection**	**Placenta previa**	**History of SGA**
-Ring chromosomes	-Toxoplasmosis	**Low-lying placenta**	
**Genetic diseases**	-Rubella	**Placental abruption**	
-Achondroplasia	-Cytomegalovirus		
-Bloom syndrome	-Herpesvirus		
**Congenital anomalies**	-Malaria		
-Potter syndrome	-Trypanosomiasis		
-Cardiac abnormalities	-HIV		
	**Nutritional status**		
	-Low prepregnancy weight		
	-Low pregnancy weight		
	**Substance use/abuse**		
	-Cigarette smoking		
	-Alcohol		
	-Illicit drugs		
	-Therapeutic drugs		

HIV, human immunodeficiency virus; SGA, small for gestational age.

Reprinted with permission from [3].

## Genetic factors influencing SGA

### GH/IGF-1 axis

#### IGF-1 gene

Transcription of the gene for *IGF-1* is mediated by the binding of pituitary GH to specific GH receptors on hepatocytes. The secretion of IGF-1 from the liver then stimulates cell growth (particularly bone) and inhibits secretion of GH from the pituitary [10]. Consequently, mutations of the *IGF-1* gene affect growth and GH secretion and have been correlated with SGA births. A homozygous partial deletion of exons 4 and 5 of *IGF-1* was observed for one patient born SGA. The mutation truncated the IGF-1 peptide sequence from 70 to 25 amino acids and was followed by an out-of-frame nonsense sequence and stop codon. In addition to growth defects, the patient suffered from bilateral sensorineural deafness and mental retardation, a feature indicating the importance of IGF-1 in central nervous system development [11]. When treated with hGH at a 0.1 U/kg dose for 4 days, no detectable IGF-1 level could be observed in the patient. However, when treated with recombinant human IGF-1 (rhIGF-1) therapy for one year (three months at 40 mcg/kg/day, nine months at 80 mcg/kg/day), insulin sensitivity, bone mineral density, and line growth of this patient were improved [12].

A second patient born SGA with sensorineural deafness and mental retardation was evaluated for *IGF-1* defects. Investigators observed a T→A transversion in the 3′-untranslated region of exon 6 that caused the expression of a truncated version of exon 6 and an altered E domain of the IGF-1 prohormone. hGH therapy for this patient (200 mcg hGH/day intramuscular doses for seven days) afforded no improvement in IGF-1 levels [13]. It should be noted that a second research group later sequenced *IGF-1* (exons 1–6) in 53 children born SGA and determined that none of the mutations in the coding region of *IGF-1* correlate with SGA stature [14].

Similarly, an *IGF-1* defect was observed for a third patient who had initially been evaluated at the age of 21 years for SGA birth size [15]. In addition to SGA size, the patient initially presented with bilateral hearing loss, microcephaly, and severe mental retardation. When investigators re-evaluated the patient at 55 years of age, unusually high serum levels of IGF-1 were noted, which varied from the patient phenotypes described by Woods and Bonapace [11-13]. Furthermore, both insulin-like growth factor binding protein-3 (IGFBP-3) and insulin-like growth factor-1 receptor (IGF-1R) levels were normal. However, by sequencing *IGF-1*, investigators detected a nucleotide substitution at position 274 (G→A) in the sequence, which caused an amino acid substitution at position 44 of the IGF-1 protein (V44M). The modified IGF-1 protein displayed a 90-fold-lower binding affinity for IGF-1R than the wild-type derivative, although the mutated protein had normal binding capacity for IGFBPs. This reduced affinity of IGF-1 for IGF-1R resulted in diminished phosphorylation of IGF-1R and downstream-acting signaling proteins, particularly Akt/PKB [16,17].

Finally, when a fourth patient born SGA in length and weight was evaluated for *IGF-1* defects, investigators discovered a homozygous G→A missense mutation in the gene that caused replacement of arginine by a glutamine at position 36 (R36Q) in the C domain of the corresponding IGF-1 protein. This change decreased the binding affinity of IGF-1 for IGF-1R by nearly three-fold, but normal affinity for IGFBP-3 was maintained [18,19]. This study confirmed that *IGF-1* mutations that lead to only partial loss of IGF-1 protein activity can cause significant postnatal as well as prenatal growth defects. While high-dose hGH therapy (400 mcg/kg/week) promoted successful catch-up growth, the patient’s treatment modality was recently changed to rhIGF-1 therapy [20]. It should be noted that rhIGF-1 therapy has only been approved by the US FDA to treat patients with severe primary IGF-1 deficiency or patients with GH gene deletions who have developed neutralizing antibodies to GH [21]. The genotype-phenotype correlations and response to hGH therapy for each of these patients expressing *IGF-1* mutations is summarized in Table 2[11,13,16-18]. 

**Table 2:** **Phenotypic characteristics and response to hGH therapy for patients with *IGF-1 *mutations**

**Genetic mutation**	**Phenotype**	**GH response**	**Ref.**
Deletion of exons 4 and 5	Birth weight −3.9 SD; birth length −5.4 SD; sensorineural deafness and mental retardation; nearly undetectable IGF-1 levels	−	Woods, 1996 [11]
Truncated version of exon 6	Birth weight −4 SD; birth length −6.5 SD; sensorineural deafness and mental retardation; low serum IGF-1 levels	−	Bonapace, 2003 [13]
V44M	Birth weight −3.9 SD score; birth length −4.3 SD score; bilateral hearing loss, microcephaly, severe mental retardation; elevated GH levels and IGF-1 levels but normal IGFBP-3 levels	n.a.	Walenkamp, 2005 [16] Denley, 2005 [17]
R36Q	Birth weight −2.5 SD score; birth length −3.7 SD score; mild mental development delay; reduced IGF-1 levels but increased IGFBP-3 levels	+	Netchine, 2006 [18]

hGH, human growth hormone; IGF-1, insulin-like growth factor-1; IGFBP-3, insulin-like growth factor binding protein-3; n.a., not available; SD, standard deviation.

#### IGF-1R

Various compound heterozygous mutations throughout the coding sequence of *IGF-1R* have been described for multiple families, with each case exhibiting phenotype variations [22]. Typically, *IGF-1R* mutations can be classified as point mutations or partial deletions. When one patient born SGA with significantly delayed postnatal growth was evaluated for *IGF-1R* mutations, investigators determined that two point mutations in exon 2 of *IGF-1R* caused two single-base pair substitutions in the codons for amino acid 108 (CGG→CAG) and 115 (AAA→AAC) of the corresponding protein. This change resulted in two-thirds-lower binding affinity of IGF-1 to IGF-1R in fibroblasts as compared with controls. When treated with hGH therapy (37.5 mcg/kg/week), the patient’s growth rate was increased to the 75th percentile for her age [23].

Similarly, a second patient born SGA who suffered from postnatal growth delay, microcephaly, and mild mental retardation was evaluated for *IGF-1R* mutations. A heterozygous point mutation CGA to TGA (Arg59Ter) in exon 2 of *IGF-1R* caused early termination of transcription of the IGF-1R protein, leading to reduced receptor expression on the cell surface, as well as decreased autophosphorylation and phosphorylation of signaling proteins [23]. When treated with hGH at 30 mcg/kg/day starting at age 6 years, the patient’s height increased by 1.01 SD after two years of therapy, indicating that hGH therapy can improve quality of life for SGA patients with this mutation [24].

When 24 children born SGA were evaluated by direct sequencing of *IGF-1R* to identify causal mutations, two patients were observed to have a heterozygous missense mutation (C→T) of *IGF-1R*, which altered the cleavage site of the proreceptor of IGF-1R from RLRR to RLQR (R709Q). This mutation inhibited the expression of mature IGF-1R from the IGF-1R precursor protein. Interestingly, the two patients who presented with this mutation had different levels of mental development. While patient 1 displayed mental retardation, patient 2 had normal intellectual development. Thus, no link exists between the heterozygous *IGF-1R* mutation and intellectual development [25].

Similarly, two more patients were evaluated and determined to present with a missense mutation in the intracellular kinase domain of *IGF-1R*. The older patient, a 35-year-old mother, showed above-average intelligence and no dysmorphic features, but her height (−4.0 SD score) and head circumference (−3.0 SD score) showed growth retardation. Her daughter, patient 2, was born SGA and showed normal mental development but delayed motor development by the age of 15 months. Both patients showed increased IGF-1 levels. Sequence analysis of *IGF-1R* showed a heterozygous G→A nucleotide substitution, which changed the amino acid sequence of IGF-1R at position 1050 from glutamic acid to lysine. This mutation did not affect expression of IGF-1R protein, but the sequence alteration reduced autophosphorylation of IGF-1R and activation of PKB/Akt [26]. Similarly, a 13.6-year-old girl who displayed short stature (−5.0 SD score) and reduced bone age (9.7 years), as well as elevated IGF-1 levels and no improvement in height following six months of treatment with hGH therapy at a daily dose of 70 mcg/kg/day, was evaluated for IGF-1R mutations. A heterozygous G→A point mutation at position 1577 of *IGF-1R* resulted in substitution of arginine with glutamine at residue 481 of the corresponding protein (R481Q). This mutation altered the α-subunit of IGF-1R, leading to reduced phosphorylation and cell growth [27]. Recently, a third report has described a similar *IGF-1R* mutation in which alanine replaced glycine at position 1125 in seven patients from the same family, causing reduced receptor autophosphorylation and phosphorylation of downstream kinases [28].

Finally, a patient born SGA with high IGF-1 levels who showed only marginal improvement in height following treatment with hGH therapy at the age of 7.4 years (doses ranging from 31 to 36 mcg/kg/day) was evaluated for *IGF-1R* mutation. Gene sequencing showed heterozygous T→A mutation at position 1886, which resulted in substitution of valine with glutamic acid at position 599 of the protein (V599E). This mutation interfered with the receptor trafficking pathway, diminishing the density of the receptor on the cell surface [29]. The genotype-phenotype correlations and response to hGH therapy for each of these patients expressing *IGF-1R* point mutations is summarized in Table 3[23,25-29]. 

**Table 3:** **Phenotypic characteristics and response to hGH therapy for patients with *IGF-1R *mutations**

**Genetic mutation**	**Phenotype**	**GH response**	**References**
R108Q K115N	Birth weight −3.5 SD score; delayed motor skill development; psychiatric anomalies; normal IGF-1 levels, delayed motor development	+	Abuzzahab, 2003 [23]
R59X	Birth weight −3.5 SD score; birth length −5.8 SD score; microcephaly, mild retardation, and delayed motor and speech development;	+	Abuzzahab, 2003 [23]
R709Q	Birth weight −1.5 SD score; birth length −1.0 SD score; significant mental retardation	N/A	Kawashima, 2005 [25]
E1050K	Birth height −0.3 SD score, birth weight −2.1 SD score; height at 35 years −4.0 SD score; head circumference at 35 years −3.0 SD score; no dysmorphic features; high IGF-1 levels	N/A	Walenkamp, 2006 [26]
R481Q	Height −4.9 SD score, reduced bone age, elevated IGF-1 levels	−	Inagaki, 2007 [27]
G1125A	Birth weight −1.7 SD score; head circumference at birth −3.7 SD score; normal mental development	N/A	Kruis, 2010 [28]
V599E	Birth weight −2.3 SD score; birth head circumference <3rd percentile; high IGF-1 levels; mental retardation	−	Wallborn, 2010 [29]

hGH, human growth hormone; IGF-1, insulin-like growth factor-1; N/A, not available; SD, standard deviation.

In addition to point mutations, distal deletions of the terminal long arm of chromosome 15 have also been linked to patients born SGA, although these mutations are quite rare. Often, these patients present with symptoms resembling Prader-Willi or Angelman syndrome, two diseases resulting from deletions in the 15q11q13 region [30]. One patient born SGA who exhibited continued growth retardation at the age of 4.5 years was evaluated for such distal deletion. It was determined that the patient presented with partial monosomy 15q26.2→15qter, correlating to a deleted critical region of approximately 5.7 Mb [31]. This deletion includes the region 15q26.3, to which the IGF-1R gene has been assigned [32]. A similar deletion was observed for a patient born SGA who displayed a heterozygous 8.58 Mb deletion in the same region [33]. Similarly, a patient born SGA who showed significant growth retardation by the age of 2 years was evaluated for deletions in chromosome 15. Results indicated that the maternally derived chromosome 15 had a 4.7 Mb deleted region, which included 15q26.2 [34]. The smallest deletion of chromosome 15 that has been observed to cause SGA birth involves a mutation in exons 11–21 of the *IGF-1R* gene (a 0.095 Mb deletion) and was associated with SGA births over three generations in a single family [35]. Typically, patients with partial deletions in this region display mental and psychomotor developmental retardations more often than patients with point *IGF-1R* mutations [22].

Fortunately, patients with partial deletions of chromosome 15 respond favorably to hGH treatment. A patient born SGA who displayed a heterozygous loss of 15q26.2→15qter began hGH treatment at the age of 5.3 years at a dose of 1 mg/m^2^/day (approximately 30 mcg/kg/day). Rapid growth catch-up was observed, and by the age of 15 years the patient had nearly reached her target height (−1.6 SD score) [36]. Similarly, two patients displaying deletions in exons 1–21 and exons 3–21 were treated with hGH therapy at a dose of 1 mg/m^2^/day (approximately 30 mcg/kg/day). For both patients, treatment resulted in moderate increase in height of approximately +1 SD after one year [37].

#### Acid-Labile Subunit (ALS) Deficiency

In serum, IGF-1 circulates in complex with IGFBP-3 or IGFBP-5 and an ALS, an 85-kDa glycoprotein that functions to prolong the half-life of the IGF-IGFBP-3/IGFBP-5 binary complex [38]. Sixteen different mutations of the *IGFALS* gene, located at 16p13.3 on chromosome 16, have been observed in patients who presented with reduced postnatal growth. The type of *IGFALS* gene mutation varies, including missense, nonsense, deletion, duplication and insertion that cause frameshift and premature stop codons, and in-frame duplication mutations, but nearly all of the mutations show autosomal recessive pattern of inheritance and cause defects in the leucine-rich repeat region of the corresponding ALS protein (Table 4) [39-47]. All of these mutations result in circulating ALS levels that are barely detectable based on enzyme-linked immunoabsorbent assay, radioimmunoassay, or Western immunoblot assays, indicating that the mutations likely inhibit the corresponding protein from being secreted by the liver or cause the protein to degrade rapidly after secretion. The circulating ALS deficiency results in a severe reduction in IGF-1 and IGFBP-3 levels, insulin insensitivity, and pubertal delay. hGH therapy was initiated for some of these patients in an effort to increase growth rate. However, despite the treatments, ranging in duration from six months to more than two years, very little growth response was observed. However, it has been suggested that hGH therapy may be beneficial for heterozygous carriers who still carry one intact *IGFALS* allele. 

**Table 4:** **Genetic mutations involved in ALS deficiency [**[39]**-**[47]**]**

**Genetic mutation**	**Type of mutation**	**Homozygous/Heterozygous**
E35KfsX87	Frameshift, premature stop codon	Homozygous
El35GfsX17	Frameshift, premature stop codon	Heterozygous
C60S	Missense	Compound heterozygous
P73L	Missense	Homozygous
L134Q	Missense	Homozygous
L172F	Missense	Homozygous
A183SfsX149	Frameshift, premature stop codon	Compound heterozygous
S195_R197dup	In-frame insertion of 3 amino acids, SLR	Compound heterozygous
L241P	Missense	Compound heterozygous
L244F	Missense	Compound heterozygous
N276S	Missense	Homozygous
Q320X	Nonsense	Homozygous
L437_L439dup	In-frame insertion of 3 amino acids, LEL	Homozygous
D440N	Missense	Homozygous
L497FfsX40	Frameshift, premature stop codon	Homozygous
C540R	Missense	Compound heterozygous

ALS, acid-labile subunit.

### Select polymorphisms

#### Obesity and diabetes

For many individuals born SGA, health concerns such as obesity, type 2 diabetes, hypertension, and ischemic heart disease are often encountered later in life [48-50]. In one study, DNA samples from 546 patients (227 children born SGA and 319 born AGA) were analyzed for 54 single nucleotide polymorphisms (SNPs) associated with diabetes or obesity. Genetic variations in five of these SNPs (*KCNJ11*, *BDNF*, *PFKP*, *PTER*, and *SEC16B*) correlated with SGA size. Therefore, genetic factors that contribute to obesity and type 2 diabetes likely correlate with SGA [51].

#### Angiotensinogen gene variants

Angiotensinogen (AGT) is an α2-globulin precursor to angiotensin II that regulates blood pressure and overall homeostasis [52]. In one study, 174 women and their 162 infants born SGA were compared with 400 women and their 240 infants born AGA. The study evaluated these individuals for a methionine to threonine substitution at codon 235 (235Met >Thr) in the *AGT* gene, a mutation associated with pregnancy complications such as preeclampsia [53]. The results showed a higher frequency of the 235Thr allele in both mothers (0.60 for SGA versus 0.36 for controls) and infants (0.59 for SGA versus 0.38 for controls) who were associated with SGA births [54]. However, the mechanism by which the 235Met >Thr mutation affects maternal-placental and fetal-placental circulation and, consequently, fetal growth is not understood. Interestingly, a prior study found no correlation between this polymorphism and an increased risk of SGA birth. The differences between the findings of the two investigations were attributed, in part, to variation in ethnic diversity between the two study groups [55].

#### Deletion of exon 3 growth hormone receptor (d3-GHR)

The *d3-GHR* polymorphism, a 2.7 kB deletion in exon 3 of the *GHR* gene, is a common genetic defect in individuals with normal height and those born SGA [56]. However, for patients born SGA, the *d3-GHR* polymorphism has been investigated as a potential mutation that affects hGH therapy due to its role in GH signaling. When response to hGH therapy was compared between children born SGA who had only full-length *GHR* versus at least one *d3-GHR* allele, results showed that patients with the *d3-GHR* polymorphism responded 1.7 to 2 times better to hGH therapy than patients with only the full-length gene [57]. Similarly, SGA patients with either two full-length *GHRs* (fl/fl) or one (d3/fl) or two (d3/d3) *d3-GHR* alleles were administered hGH for 12 months at a mean dose of 56 ± 11 mcg/kg/day. At the end of 12 months, carriers of either one or two *d3-GHR* alleles were observed to respond slightly better to hGH therapy than patients with two full-length alleles, although the difference was not statistically significant. The authors suggested that response to hGH therapy for patients with this mutation depends on the specific causes of short stature, such as IGF-1 insensitivity or IGF-1 deficiency [58]. Consequently, children born SGA with the *d3-GHR* mutation appear to be prime candidates for hGH therapy, although these results are still controversial.

For instance, a comparison was made between the *GHR* genotype (ie, fl/fl, d3/fl, or d3/d3) of patients with GH deficiency and the individual’s response to hGH treatment. Patients were treated with hGH at a mean dose of 0.2 mg/kg/week for one year and then evaluated for height SD score, height velocity, and height velocity SD score. No statistically significant difference with respect to the measured outcomes could be observed between the patients with the *d3-GHR* allele and patients who were homozygous for the full-length *GHR*. Furthermore, this study observed that there was no relationship between an individual’s baseline phenotype and his/her *GHR* genotype, suggesting that the *d3-GHR* allele does not affect height in GH deficiency [59]. This lack of correlation between *d3-GHR* genotype and response to hGH treatment was also confirmed in studies for patients born SGA [60,61].

Recently, a meta-analysis of 15 studies investigating the effects of *d3-GHR* genotype and a patient’s first-year response to hGH therapy, including height gain and change in growth velocity, was conducted. The results of this analysis indicated that patients with the *d3-GHR* allele showed improved growth velocity when treated with hGH therapy, but the treatment outcome was affected by the dose (low doses of hGH showed best response) and age at time of treatment (older patients responded more favorably). It should be noted, however, that this meta-analysis did not discriminate with respect to the cause of short stature [62]. In a recent 3-year review, Doerr et al conclude that the determination of GHR isoforms for deletion of exon 3 is not particularly useful in defining the overall response to GH in short SGA children [63].

### Uniparental disomy (UPD) and imprinting effects

UPD is a process whereby a person inherits two copies of a gene or chromosome from one parent and no copies from the other parent. In most cases, UPD does not affect fetal development. However, if a UPD gene is also an imprinted gene, there may be adverse effects to the fetus, because UPD of imprinted genes is equivalent to functional nullisomy [64]. The transcriptional regulation of imprinted genes varies from normal genes in that imprinted genes are only active from one parent allele. For instance, a gene may be active only when paternally inherited; the maternal allele of this gene is “switched off.” Conversely, imprinted genes can be maternally expressed and paternally imprinted [65]. Thus, if a patient inherits two versions of an imprinted gene (eg, two copies of a maternal, “switched-off” gene), phenotype abnormalities may result. Studies have indicated that several UPDs can be responsible for short stature in patients born SGA.

#### SRS

SRS is a disorder characterized by reduced birth weight, facial features including triangular shape and pointed chin, and body asymmetry [66,67]. Growth restrictions continue through life and often correlate with fasting hypoglycemia [68]. hGH treatment, given daily as subcutaneous injections at a dose of 35 mcg/kg/day for up to three years, is usually suggested for these patients [69].

The genetic causes of SRS vary, with cases of autosomal-dominant, autosomal-recessive, and X-linked inheritance all observed (as reviewed by Hitchins and Abu-Amero) [68,70]. However, the most referenced causal candidates for this disease involve mutations on chromosomes 7 and 11, which both contain groups of genes that undergo genomic imprinting [68]. Since the early 1990s, maternal uniparental disomy 7 (mUPD7), both full mUPD7 and mUPD for the long arm of chromosome 7, were documented to be the cause of SRS in approximately 10% of cases [71]. However, the phenotype of an SRS patient presenting UPD7 cannot be predicted, as the exact etiology of the mutation varies [72]. Polymerase chain reaction with microsatellite repeat markers or Southern blot analysis with variable number of tandem repeats can effectively be used to screen patients for mUPD7 [73].

**Figure 1:**
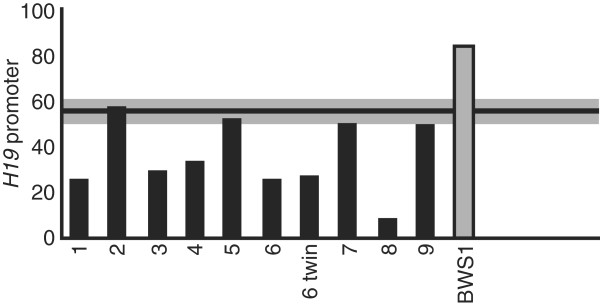
**Quantitative representation of methylation indices for *H19-IGF2 *ICR1 in individuals with SRS (individuals 1–9 and individual 6’s twin) and an individual with isolated hypermethylation of the *H19 *promoter (BWS1).** Five individuals with SRS displayed partial loss of *H19-IGF2* ICR1, indicated by the bar below the shaded area near 50%. SRS, Silver-Russell syndrome. Reprinted from [74] with permission from Macmillan Publishers Ltd; copyright 2005.

In addition to mUPD7, the imprinted region on chromosome 11p15 has been associated with SRS in up to 65% of patients. Specifically, hypomethylation at the imprinting center region 1 (ICR1) was associated with fetal growth retardation in SRS patients (Figure 1) [74,75]. Generally, the ICR1 region regulates the expression of *IGF2* and *H19*, and loss of methylation of this region is associated with approximately 50% of SRS cases [68]. However, an inherited duplication (0.76 – 1 Mb) in the ICR2 domain of 11p15 has also been shown to be involved in the etiology of SRS. The duplicated region included the maternally expressed genes *KCNQ1*, *CDKN1C*, *TSSC5/SLC22A8* and *TSSC3/PHDLA2* and the paternally expressed gene *LIT1*[76]. It should be noted, however, that the distribution of methylation values among patients with SRS is quite varied, making clinical diagnosis of the disease based on methylation analysis difficult [77]. In general, use of hGH has become a standard treatment regimen for patients with SRS, despite the limited number of evaluations regarding the effectiveness of this treatment [78]. 

#### mUPD

UPD of the long arm of chromosome 14 (UPD14) has been associated with both below-average growth and mental retardation. Initially, it was not known whether the congenital anomalies present in UPD14 patients resulted from an extra copy of an active imprinted gene (ie, two genes that were “switched on”) or the absence of gene expression caused by the presence of two repressed alleles (ie, two genes “switched off”). To determine the likely cause of the phenotype, patients with distal partial trisomy for chromosome 14 (Ts14) were evaluated to determine genotype-phenotype correlations to determine whether the partial trisomy was of maternal or paternal origin. By investigating patients with an extra copy of either maternally inherited or paternally inherited copies of chromosome 14, the investigators hoped to observe more pronounced effects of the disease if it was caused by active imprinted genes. All 13 patients with distal maternal Ts14 (mTs14) were born SGA. Conversely, over half of the patients with paternal Ts14 (pTs14) were born at weights AGA, indicating that an absence of paternal information likely causes growth retardation in patients with UPD14. The minimum trisomic regions 14q31.1-14qter and 14q24.3-14qter were identified as possibly containing the imprinted genes [79]. Overall, the phenotype of patients with mUPD14 can be quite variable. A review of 24 cases of patients displaying mUPD14 attributes the growth retardation of these patients to confined placental mosaicism and imprinted genes that cause early skeletal maturation, although unusual phenotypes may also be caused by autosomal, recessively inherited mutations [80].

## hGH treatment for SGA

Much research has correlated genetic mutations with SGA births, but the ability to predict the effectiveness of hGH therapy for each mutation remains controversial. Table 5 summarizes the various mutations that have been shown to cause SGA and the likelihood that hGH therapy will promote growth for individuals with these mutations [11,13,16-18,23,25-29,39-47,51,53-55,57-62,68,70,74-76,78-80]. Some patients with *IGF-1* mutations have shown positive growth catch-up when treated with hGH therapy, while others have shown better response to rhIGF-1 therapy. Alternatively, the response to hGH therapy for patients with *IGF-1R* mutations appears to correlate with the type of mutation; patients with distal deletions of the *IGF-1R* gene generally have improved GH-induced catch-up growth as compared with patients who have *IGF-1R* point mutations. Finally, the ability to predict the effectiveness of hGH treatment depending on the specific disease (eg, children with SRS versus children with UPD14) has not been thoroughly reviewed, possibly because a significant number of patients born SGA who undergo hGH therapy are never genetically diagnosed. However, despite the controversies, clinical studies have successfully elucidated some trends about hGH treatment on growth in children born SGA (as reviewed by Simon et al [81] and Saenger et al [1]). 

**Table 5:** **Summary of known genetic causes of SGA and the correlating response to hGH therapy [**[11]**,**[13]**,**[16]**-**[18]**,**[23]**,**[25]**-**[29]**,**[39]**-**[47]**,**[51]**,**[53]**-**[55]**,**[57]**-**[62]**,**[68]**,**[70]**,**[74]**-**[76]**,**[78]**-**[80]**]**

**Class of genetic mutation**	**Specific genetic variant**	**Response to hGH therapy**
	*IGF-1*	Generally not effective
	*IGF-1R*	Good for partial distal deletions; generally not effective for point mutations
GH/IGF-1 axis	Point	
	Distal	
	ALS deletions	Good outcome for heterozygous carriers
	Obesity/diabetes-related genes	Unclear
Select Polymorphisms	Angiotensinogen gene	Unclear
	*d3-GHR*	Good outcome, but dose and age matter
	SRS	hGH therapy is commonly used for SRS, but correlation between effectiveness and specific genetic mutation has not been carefully evaluated
	Full mUPD7	
UPD/imprinting effects	mUPD7 for long arm of chromosome 7	
	Hypomethylation at ICR1 on 11p15	
	Duplication of ICR2 on 11p15	
	UPD14	Unclear

ALS, acid-labile subunit; ICR, imprinting control region; IGF-1, insulin-like growth factor-1; d3-GHR, deletion of exon 3 growth hormone receptor; hGH, human growth hormone; SGA, small for gestational age; SRS, Silver-Russell syndrome; UPD, uniparental disomy.

**Figure 2:**
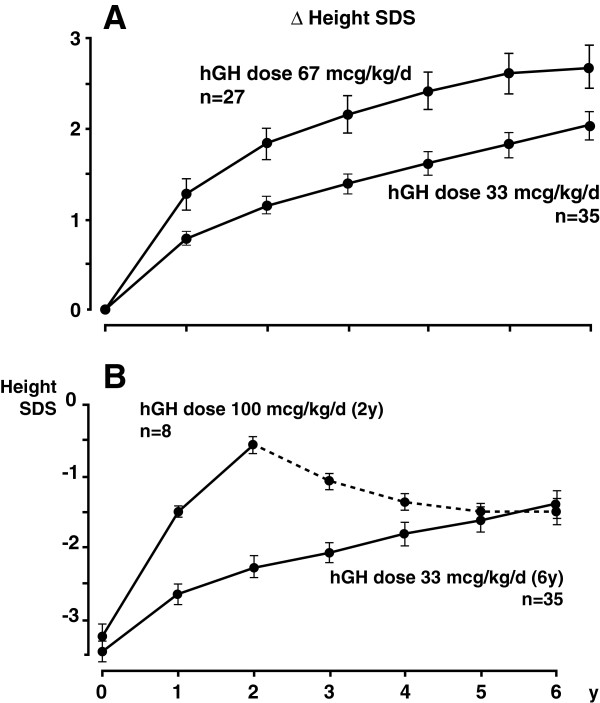
**(A) Amount of time required to increase height SD score by 2 was 2.5 years for hGH therapy administered at a dose of 67 mcg/kg/day and 5.5 years for hGH therapy administered at a dose of 33 mcg/kg/day in children born SGA.** Figure 2A reprinted with permission from [82]. (**B**) After 6 years, similar height SD scores were achieved using 2 years of high-dose (100 mcg/kg/day) hGH therapy and 6 years of low-dose (33 mcg/kg/day) hGH therapy for children born SGA. hGH, human growth hormone; SD, standard deviation; SGA, small for gestational age. Figure 2B reprinted with permission from [82].

The rate of catch-up growth promoted by hGH therapy in patients born SGA correlates with the dose; higher doses typically afford rapid height increase, although a similar response can be achieved using lower doses for a longer time. For instance, a height gain of 2 SD was achieved for patients born SGA using doses of either 67 mcg/kg/day over 2.5 years or 33 mcg/kg/day over 5.5 years (Figure 2A) [82]. However, the low-dose regimen requires three times as many injections and 50% more hGH overall than the high-dose method. The method of administration of hGH therapy can also affect height gain, though less significantly than dose. Patients who received discontinuous high-dose hGH therapy (67 mcg/kg/day for one or two years) have shown slightly increased height gain compared with patients receiving a continuous low-dose regimen (33 mcg/kg/day doses for three or four years), although discontinuation of the treatment typically corresponds with reduction in growth velocity [83]. A similar trend was observed previously by De Zeghers et al, who found that after six years, height SD scores were similar for high-dose hGH course for two years and continuous low-dose hGH treatment for six years (Figure 2B) [82]. 

In addition to the dose and method of administration, the age of initiation of hGH therapy significantly affects the outcome. Patients treated before the onset of puberty achieve optimal results. A recent study showed that children treated for one year with hGH therapy before the age of 4 years achieved greater height gain (1.7 SD score, 12.5 cm) than those treated after 4 years of age (1.2 SD score) [84]. Even among older patients, this trend persists. Patients receiving hGH therapy more than two years before puberty showed increased height gain (1.7 SD, ~12 cm) compared with patients treated fewer than two years before puberty (0.9 SD gain, 6 cm). However, nearly 90% of these patients achieved adult height within the normal range [85]. Conversely, patients treated during puberty achieved height gain of only 0.6 SD score, and fewer than 50% of these patients achieved normal adult height [86].

In 2003, Ranke et al developed a model that essentially summarized the trends that we have described and that could be used by physicians to individualize hGH treatment for SGA patients. Using a pharmacoepidemiological survey of 613 children, various trends were elucidated. In fact, the model could be used to explain approximately 50% of the variability associated with hGH therapy response during the first and second years of treatment. Nearly 35% of the variability could be attributed to the dose, followed by the patient’s age at the start of treatment. Subsequent growth during the second year of treatment could be predicted based on a successful first year of treatment [87].

It must be mentioned, though not stressed, that some controversy regarding the use of hGH arose in 2011 due to results from a study conducted in France, the Santé Adulte GH Enfant (SAGhE) study. The results from this study indicated that long-term use of hGH in children with short stature could increase a patient’s risk of death [88]. The SAGhE study reported that hGH therapy, when administered to patients at doses above 50 mcg/kg/day, increased the risk of death by 30% as compared to the general population in France. This effect was attributed to an increased likelihood of bone-tumor formation, cardiovascular disease, and cerebrovascular events. These results concerned patients born SGA, as the normal recommended dose of hGH therapy can be approximately 70 mcg/kg/day (Table 6) [1]. However, recent publications and an FDA report have noted flaws associated with the SAGhE study design [88-90]. In many other long-term evaluations of large groups of patients undergoing hGH therapy, the overall safety profile is favorable [91-95]. No increased risk of death due to leukemia, cancer, or cardiovascular disorders was observed. 

**Table 6:** Use of hGH therapy in SGA children in the United States and Europe

	**FDA-approved indication in 2001**	**EMEA-approved indication in 2003**
Age at start of treatment (year)	2	4
Height SDS at start	Not stated	−2.5 SD
Growth velocity before treatment	No catch-up growth	Less than 0 SD for age
Reference to midparental height	Not stated	Height SDS > 1 SD below midparental height SDS
Dose (mcg/kg/day)	70	35

FDA, United States Food and Drug Administration; EMEA, European Agency for the Evaluation of Medicinal Products; hGH, human growth hormone; SDS, standard deviation score; SGA, small for gestational age.

Reprinted with permission from [1].

## Conclusions

Based on results from more than 20 years of research, numerous genetic causes for SGA births have been realized. Genetic defects in either *IGF-1* or *IGF-1R* that result in SGA size typically correlate with phenotypical features such as microcephaly and mental retardation. The most predictive factors for *IGF-1R* deletion include small birth size, head size, and stature, as well as high IGF-1 levels, developmental delay, and micrognathia. hGH therapy in patients with mutations in *IGF-1* has shown moderate success. Furthermore, for patients with *IGF-1R* mutations, hGH treatment has been shown to be especially promising, particularly for those with distal deletions of the terminal long arm of chromosome 15. Overall, in studies in which the genotype of SGA patients was not known and hGH therapy was conducted, improvements were observed for most of the patient population, particularly if therapy was begun at a young age.

However, despite these positive results, a number of questions regarding the effectiveness of the treatment remain. For instance, hGH therapy for children with SRS has shown positive results, but overall the improvements are often not statistically significant. Furthermore, the differences in SGA patient response to hGH therapy are still only slightly understood. While much of the diversity in response rates to hGH therapy for SGA patients correlates with the type of genetic mutation, the role of additional factors, such as ethnicity, on this treatment still requires significant research.

## Abbreviations

(AGA): Appropriate for gestational age; (AGT): Angiotensinogen; (ALS): Acid-labile subunit; (FDA): Food and Drug Administration; (GH): Growth hormone; (ICR1): Imprinting center region 1; (IGFBP-3): Insulin-like growth factor binding protein-3; (IGF): Insulin-like growth factor; (IGF-1R): Insulin-like growth factor-1 receptor; (mUPD7): Maternal uniparental disomy 7; (hGH): Recombinant human GH; (rhIGF-1): Recombinant human IGF-1; (SAGhE): Santé Adulte GH Enfant study; (SGA): Small for gestational age; (SD): Standard deviation; (SRS): Silver-Russell syndrome; (Ts14): Trisomy for chromosome 14; (UPD): Uniparental disomy; (US): United States.

## Competing interests

Dr. Saenger reports that he receives grant support from Novo Nordisk Inc., and that he is a consultant for LG and Biopartners. Dr. Reiter reports that he has received payment from Novo Nordisk Inc. for board membership; from Abbott Pharmaceuticals as a consultant; from Quintiles for development of educational presentations, and from various pharmaceutical companies for lectures, including service on speakers bureaus.

## Authors’ contributions

The authors contributed equally to this work and were involved in the development of its concept, outline, and narrative. At all stages, the authors discussed the data presented and commented on the manuscript. Both authors read and approved the final manuscript.

## References

[B1] Saenger P Czernichow P Hughes I Reiter EO Small for gestational age: short stature and beyond Endocr Rev 2007 28 2 219 251 17322454 10.1210/er.2006-0039

[B2] Clayton PE Cianfarani S Czernichow P Johannsson G Rapaport R Rogol A Management of the child born small for gestational age through to adulthood: a consensus statement of the International Societies of Pediatric Endocrinology and the Growth Hormone Research Society J Clin Endocrinol Metab 2007 92 3 804 810 17200164 10.1210/jc.2006-2017

[B3] Lee PA Chernausek SD Hokken-Koelega AC Czernichow P International Small for Gestational Age Advisory Board consensus development conference statement: management of short children born small for gestational age, April 24-October 1, 2001 Pediatrics 2003 111 6 Pt 1 1253 1261 12777538 10.1542/peds.111.6.1253

[B4] Arya AD Small for gestation and growth hormone therapy Indian J Pediatr 2006 73 1 73 78 10.1007/BF02758265 16444066

[B5] Rapaport R Tuvemo T Growth and growth hormone in children born small for gestational age Acta Paediatr 2005 94 10 1348 1355 10.1080/08035250510043860 16263627

[B6] Hediger ML Overpeck MD Maurer KR Kuczmarski RJ McGlynn A Davis WW Growth of infants and young children born small or large for gestational age: findings from the Third National Health and Nutrition Examination Survey Arch Pediatr Adolesc Med 1998 152 12 1225 1231 9856434 10.1001/archpedi.152.12.1225

[B7] Karlberg J Albertsson-Wikland K Growth in full-term small-for-gestational-age infants: from birth to final height Pediatr Res 1995 38 5 733 739 10.1203/00006450-199511000-00017 8552442

[B8] Hokken-Koelega AC De Ridder MA Lemmen RJ Den Hartog H De Muinck Keizer-Schrama SM Drop SL Children born small for gestational age: do they catch up? Pediatr Res 1995 38 2 267 271 10.1203/00006450-199508000-00022 7478827

[B9] The Growth Genetics Consortium. Available at http://www.growthgeneticsconsortium.org/index.html Accessed February 15, 2012.

[B10] Le Roith D Seminars in medicine of the Beth Israel Deaconess Medical Center. Insulin-like growth factors N Engl J Med 1997 336 9 633 640 10.1056/NEJM199702273360907 9032050

[B11] Woods KA Camacho-Hubner C Savage MO Clark AJ Intrauterine growth retardation and postnatal growth failure associated with deletion of the insulin-like growth factor I gene N Engl J Med 1996 335 18 1363 1367 10.1056/NEJM199610313351805 8857020

[B12] Woods KA Camacho-Hubner C Bergman RN Barter D Clark AJ Savage MO Effects of insulin-like growth factor I (IGF-I) therapy on body composition and insulin resistance in IGF-I gene deletion J Clin Endocrinol Metab 2000 85 4 1407 1411 10.1210/jc.85.4.1407 10770174

[B13] Bonapace G Concolino D Formicola S Strisciuglio P A novel mutation in a patient with insulin-like growth factor 1 (IGF1) deficiency J Med Genet 2003 40 12 913 917 10.1136/jmg.40.12.913 14684690 PMC1735341

[B14] Coutinho DC Coletta RR Costa EM Pachi PR Boguszewski MC Damiani D Mendonca BB Arnhold IJ Jorge AA Polymorphisms identified in the upstream core polyadenylation signal of IGF1 gene exon 6 do not cause pre- and postnatal growth impairment J Clin Endocrinol Metab 2007 92 12 4889 4892 10.1210/jc.2007-1661 17895313

[B15] van Gemund JJ Laurent de Angulo MS van Gelderen HH Familial prenatal dwarfism with elevated serum immuno-reactive growth hormone levels and end-organ unresponsiveness Maandschr Kindergeneeskd 1970 37 11 372 382 5438864

[B16] Walenkamp MJ Karperien M Pereira AM Hilhorst-Hofstee Y van Doorn J Chen JW Mohan S Denley A Forbes B van Duyvenvoorde HA van Thiel SW Sluimers CA Bax JJ de Laat JA Breuning MB Romijn JA Wit JM Homozygous and heterozygous expression of a novel insulin-like growth factor-I mutation J Clin Endocrinol Metab 2005 90 5 2855 2864 10.1210/jc.2004-1254 15769976

[B17] Denley A Wang CC McNeil KA Walenkamp MJ van DH Wit JM Wallace JC Norton RS Karperien M Forbes BE Structural and functional characteristics of the Val44Met insulin-like growth factor I missense mutation: correlation with effects on growth and development Mol Endocrinol 2005 19 3 711 721 15576456 10.1210/me.2004-0409

[B18] Netchine I Azzi S Houang M Seurin D Perin L Ricort JM Daubas C Legay C Mester J Herich R Godeau F Le Bouc Y Partial IGF-1 deficiency demonstrates the critical role of IGF-1 in growth and brain development. [abstract] Horm Res 2006 65 suppl 4 29 10.1210/jc.2009-0452 19773405

[B19] Netchine I Azzi S Houang M Seurin D Perin L Ricort JM Daubas C Legay C Mester J Herich R Godeau F Le Bouc Y Partial primary deficiency of insulin-like growth factor (IGF)-I activity associated with IGF1 mutation demonstrates its critical role in growth and brain development J Clin Endocrinol Metab 2009 94 10 3913 3921 10.1210/jc.2009-0452 19773405

[B20] Netchine I Azzi S Le Bouc Y Savage MO IGF1 molecular anomalies demonstrate its critical role in fetal, postnatal growth and brain development Best Pract Res Clin Endocrinol Metab 2011 25 1 181 190 10.1016/j.beem.2010.08.005 21396584

[B21] Graul AI Prous JR The year’s new drugs Drug News Perspect 2006 19 1 33 53 16550255

[B22] Klammt J Kiess W Pfaffle R IGF1R mutations as cause of SGA Best Pract Res Clin Endocrinol Metab 2011 25 1 191 206 10.1016/j.beem.2010.09.012 21396585

[B23] Abuzzahab MJ Schneider A Goddard A Grigorescu F Lautier C Keller E Kiess W Klammt J Kratzsch J Osgood D Pfaffle R Raile K Seidel B Smith RJ Chernausek SD IGF-I receptor mutations resulting in intrauterine and postnatal growth retardation N Engl J Med 2003 349 23 2211 2222 10.1056/NEJMoa010107 14657428

[B24] Raile K Klammt J Schneider A Keller A Laue S Smith R Pfaffle R Kratzsch J Keller E Kiess W Clinical and functional characteristics of the human Arg59Ter insulin-like growth factor i receptor (IGF1R) mutation: implications for a gene dosage effect of the human IGF1R J Clin Endocrinol Metab 2006 91 6 2264 2271 10.1210/jc.2005-2146 16569742

[B25] Kawashima Y Kanzaki S Yang F Kinoshita T Hanaki K Nagaishi J Ohtsuka Y Hisatome I Ninomoya H Nanba E Fukushima T Takahashi S Mutation at cleavage site of insulin-like growth factor receptor in a short-stature child born with intrauterine growth retardation J Clin Endocrinol Metab 2005 90 8 4679 4687 10.1210/jc.2004-1947 15928254

[B26] Walenkamp MJ van der Kamp HJ Pereira AM Kant SG van Duyvenvoorde HA Kruithof MF Breuning MH Romijn JA Karperien M Wit JM A variable degree of intrauterine and postnatal growth retardation in a family with a missense mutation in the insulin-like growth factor I receptor J Clin Endocrinol Metab 2006 91 8 3062 3070 10.1210/jc.2005-1597 16757531

[B27] Inagaki K Tiulpakov A Rubtsov P Sverdlova P Peterkova V Yakar S Terekhov S LeRoith D A familial insulin-like growth factor-I receptor mutant leads to short stature: clinical and biochemical characterization J Clin Endocrinol Metab 2007 92 4 1542 1548 10.1210/jc.2006-2354 17264177

[B28] Kruis T Klammt J Galli-Tsinopoulou A Wallborn T Schlicke M Muller E Kratzsch J Korner A Odeh R Kiess W Pfaffle R Heterozygous mutation within a kinase-conserved motif of the insulin-like growth factor I receptor causes intrauterine and postnatal growth retardation J Clin Endocrinol Metab 2010 95 3 1137 1142 10.1210/jc.2009-1433 20103656

[B29] Wallborn T Wuller S Klammt J Kruis T Kratzsch J Schmidt G Schlicke M Muller E van de Leur HS Kiess W Pfaffle R A heterozygous mutation of the insulin-like growth factor-I receptor causes retention of the nascent protein in the endoplasmic reticulum and results in intrauterine and postnatal growth retardation J Clin Endocrinol Metab 2010 95 5 2316 2324 10.1210/jc.2009-2404 20357178

[B30] Knoll JH Nicholls RD Magenis RE Graham JM Jr Lalande M Latt SA Angelman and Prader-Willi syndromes share a common chromosome 15 deletion but differ in parental origin of the deletion Am J Med Genet 1989 32 2 285 290 10.1002/ajmg.1320320235 2564739

[B31] Pinson L Perrin A Plouzennec C Parent P Metz C Collet M Le Bris MJ Douet-Guilbert N Morel F de Braekeleer M Detection of an unexpected subtelomeric 15q26.2 – > qter deletion in a little girl: clinical and cytogenetic studies Am J Med Genet A 2005 138A 2 160 165 10.1002/ajmg.a.30939 16114049

[B32] Peoples R Milatovich A Francke U Hemizygosity at the insulin-like growth factor I receptor (IGF1R) locus and growth failure in the ring chromosome 15 syndrome Cytogenet Cell Genet 1995 70 3–4 228 234 7789178 10.1159/000134040

[B33] Choi JH Kang M Kim GH Hong M Jin HY Lee BH Park JY Lee SM Seo EJ Yoo HW Clinical and functional characteristics of a novel heterozygous mutation of the IGF1R gene and IGF1R haploinsufficiency due to terminal 15q26.2- > qter deletion in patients with intrauterine growth retardation and postnatal catch-up growth failure J Clin Endocrinol Metab 2011 96 1 E130 E134 10.1210/jc.2010-1789 20962017

[B34] Rujirabanjerd S Suwannarat W Sripo T Dissaneevate P Permsirivanich W Limprasert P De novo subtelomeric deletion of 15q associated with satellite translocation in a child with developmental delay and severe growth retardation Am J Med Genet A 2007 143 3 271 276 17236205 10.1002/ajmg.a.31581

[B35] Veenma DC Eussen HJ Govaerts LC de Kort SW Odink RJ Wouters CH Hokken-Koelega AC de Klein A Phenotype-genotype correlation in a familial IGF1R microdeletion case J Med Genet 2010 47 7 492 498 10.1136/jmg.2009.070730 19955558

[B36] Walenkamp MJ de Muinck Keizer-Schrama SM de Mos M Kalf ME van Duyvenvoorde HA Boot AM Kant SG White SJ Losekoot M den Dunnen JT Karperien M Wit JM Successful long-term growth hormone therapy in a girl with haploinsufficiency of the insulin-like growth factor-I receptor due to a terminal 15q26.2->qter deletion detected by multiplex ligation probe amplification J Clin Endocrinol Metab 2008 93 6 2421 2425 10.1210/jc.2007-1789 18349070

[B37] Ester WA van Duyvenvoorde HA de Wit CC Broekman AJ Ruivenkamp CA Govaerts LC Wit JM Hokken-Koelega AC Losekoot M Two short children born small for gestational age with insulin-like growth factor 1 receptor haploinsufficiency illustrate the heterogeneity of its phenotype J Clin Endocrinol Metab 2009 94 12 4717 4727 10.1210/jc.2008-1502 19864454

[B38] Boisclair YR Rhoads RP Ueki I Wang J Ooi GT The acid-labile subunit (ALS) of the 150kDa IGF-binding protein complex: an important but forgotten component of the circulating IGF system J Endocrinol 2001 170 1 63 70 10.1677/joe.0.1700063 11431138

[B39] Domene HM Hwa V Jasper HG Rosenfeld RG Acid-labile subunit (ALS) deficiency Best Pract Res Clin Endocrinol Metab 2011 25 1 101 113 10.1016/j.beem.2010.08.010 21396577

[B40] Domene HM Bengolea SV Martinez AS Ropelato MG Pennisi P Scaglia P Heinrich JJ Jasper HG Deficiency of the circulating insulin-like growth factor system associated with inactivation of the acid-labile subunit gene N Engl J Med 2004 350 6 570 577 10.1056/NEJMoa013100 14762184

[B41] Domene HM Scaglia PA Lteif A Mahmud FH Kirmani S Frystyk J Bedecarras P Gutierrez M Jasper HG Phenotypic effects of null and haploinsufficiency of acid-labile subunit in a family with two novel IGFALS gene mutations J Clin Endocrinol Metab 2007 92 11 4444 4450 10.1210/jc.2007-1152 17726072

[B42] Heath KE Argente J Barrios V Pozo J az-Gonzalez F Martos-Moreno GA Caimari M Gracia R Campos-Barros A Primary acid-labile subunit deficiency due to recessive IGFALS mutations results in postnatal growth deficit associated with low circulating insulin growth factor (IGF)-I, IGF binding protein-3 levels, and hyperinsulinemia J Clin Endocrinol Metab 2008 93 5 1616 1624 10.1210/jc.2007-2678 18303074

[B43] van Duyvenvoorde HA Kempers MJ Twickler TB van Doorn J Gerver WJ Noordam C Losekoot M Karperien M Wit JM Hermus AR Homozygous and heterozygous expression of a novel mutation of the acid-labile subunit Eur J Endocrinol 2008 159 2 113 120 10.1530/EJE-08-0081 18463107

[B44] Fofanova-Gambetti OV Hwa V Kirsch S Pihoker C Chiu HK Hogler W Cohen LE Jacobsen C Derr MA Rosenfeld RG Three novel IGFALS gene mutations resulting in total ALS and severe circulating IGF-I/IGFBP-3 deficiency in children of different ethnic origins Horm Res 2009 71 2 100 110 10.1159/000183899 19129715

[B45] David A Rose SJ Miraki-Moud F Metherell LA Savage MO Clark AJ Camacho-Hubner C Acid-labile subunit deficiency and growth failure: description of two novel cases Horm Res Paediatr 2010 73 5 328 334 10.1159/000308164 20389102 PMC2868526

[B46] Bang P Fureman A-L Nilsson A-L Bostrom J Berit K Ekstrom K Hwa V Grosenfeld R Carlsson-Skwirut C A novel missense mutation of the ALSIGF gene causing a L172F substitution in LRR6 is associated with short stature in two Swedish children homozygous or compound heterozygous for the mutation Horm Res 2009 72 suppl 3 86

[B47] Gallego-Gomez E Sanchez del Pozo J Cruz Rojo J Zurita-Munoz O Gracia-Bouthelier R Heath KE Campos-Barros A Novel compound heterozygous IGFALS mutation associated with impaired postnatal growth and low circulating IGF-I and IGFBP-3 levels Horm Res 2009 72 suppl 3 90 91

[B48] Hales CN Barker DJ Clark PM Cox LJ Fall C Osmond C Winter PD Fetal and infant growth and impaired glucose tolerance at age 64 BMJ 1991 303 6809 1019 1022 10.1136/bmj.303.6809.1019 1954451 PMC1671766

[B49] Barker DJ Hales CN Fall CH Osmond C Phipps K Clark PM Type 2 (non-insulin-dependent) diabetes mellitus, hypertension and hyperlipidaemia (syndrome X): relation to reduced fetal growth Diabetologia 1993 36 1 62 67 10.1007/BF00399095 8436255

[B50] Parsons TJ Power C Manor O Fetal and early life growth and body mass index from birth to early adulthood in 1958 British cohort: longitudinal study BMJ 2001 323 7325 1331 1335 10.1136/bmj.323.7325.1331 11739217 PMC60670

[B51] Morgan AR Thompson JM Murphy R Black PN Lam WJ Ferguson LR Mitchell EA Obesity and diabetes genes are associated with being born small for gestational age: results from the Auckland Birthweight Collaborative study BMC Med Genet 2010 11 125 10.1186/1471-2350-11-125 20712903 PMC2928774

[B52] Gardes J Bouhnik J Clauser E Corvol P Menard J Role of angiotensinogen in blood pressure homeostasis Hypertension 1982 4 2 185 189 10.1161/01.HYP.4.2.185 7068178

[B53] Ward K Hata A Jeunemaitre X Helin C Nelson L Namikawa C Farrington PF Ogasawara M Suzumori K Tomoda S Berrebi S Sasaki M Corvol P Lifton RP Lalouel JM A molecular variant of angiotensinogen associated with preeclampsia Nat Genet 1993 4 1 59 61 10.1038/ng0593-59 8513325

[B54] Zhang XQ Varner M Dizon-Townson D Song F Ward K A molecular variant of angiotensinogen is associated with idiopathic intrauterine growth restriction Obstet Gynecol 2003 101 2 237 242 10.1016/S0029-7844(02)02512-7 12576245

[B55] Tower C Chappell S Kalsheker N Baker P Morgan L Angiotensinogen gene variants and small-for-gestational-age infants BJOG 2006 113 3 335 339 10.1111/j.1471-0528.2005.00841.x 16487207

[B56] Pantel J Machinis K Sobrier ML Duquesnoy P Goossens M Amselem S Species-specific alternative splice mimicry at the growth hormone receptor locus revealed by the lineage of retroelements during primate evolution J Biol Chem 2000 275 25 18664 18669 10.1074/jbc.M001615200 10764769

[B57] Dos Santos C Essioux L Teinturier C Tauber M Goffin V Bougneres P A common polymorphism of the growth hormone receptor is associated with increased responsiveness to growth hormone Nat Genet 2004 36 7 720 724 10.1038/ng1379 15208626

[B58] Binder G Baur F Schweizer R Ranke MB The d3-growth hormone (GH) receptor polymorphism is associated with increased responsiveness to GH in Turner syndrome and short small-for-gestational-age children J Clin Endocrinol Metab 2006 91 2 659 664 16291706 10.1210/jc.2005-1581

[B59] Blum WF Machinis K Shavrikova EP Keller A Stobbe H Pfaeffle RW Amselem S The growth response to growth hormone (GH) treatment in children with isolated GH deficiency is independent of the presence of the exon 3-minus isoform of the GH receptor J Clin Endocrinol Metab 2006 91 10 4171 4174 10.1210/jc.2006-0063 16868057

[B60] Carrascosa A Esteban C Espadero R Fernandez-Cancio M Andaluz P Clemente M Audi L Wollmann H Fryklund L Parodi L The d3/fl-growth hormone (GH) receptor polymorphism does not influence the effect of GH treatment (66 microg/kg per day) or the spontaneous growth in short non-GH-deficient small-for-gestational-age children: results from a two-year controlled prospective study in 170 Spanish patients J Clin Endocrinol Metab 2006 91 9 3281 3286 10.1210/jc.2006-0685 16804042

[B61] Carrascosa A Audi L Esteban C Fernandez-Cancio M Andaluz P Gussinye M Clemente M Yeste D Albisu MA Growth hormone (GH) dose, but not exon 3-deleted/full-length GH receptor polymorphism genotypes, influences growth response to two-year GH therapy in short small-for-gestational-age children J Clin Endocrinol Metab 2008 93 1 147 153 17925340 10.1210/jc.2007-1182

[B62] Wassenaar MJ Dekkers OM Pereira AM Wit JM Smit JW Biermasz NR Romijn JA Impact of the exon 3-deleted growth hormone (GH) receptor polymorphism on baseline height and the growth response to recombinant human GH therapy in GH-deficient (GHD) and non-GHD children with short stature: a systematic review and meta-analysis J Clin Endocrinol Metab 2009 94 10 3721 3730 10.1210/jc.2009-0425 19584188

[B63] Dörr HG Bettendorf M Hauffa BP Mehls O Rohrer T Stahnke N Pfäffle R Ranke MB Different relationships between the first 2 years on growth hormone treatment and the d3-growth hormone receptor polymorphism in short small-for-gestational-age (SGA) children Clin Endocrinol (Oxf) 2011 75 5 656 660 10.1111/j.1365-2265.2011.04104.x 21623854

[B64] Hoffmann K Heller R Uniparental disomies 7 and 14 Best Pract Res Clin Endocrinol Metab 2011 25 1 77 100 10.1016/j.beem.2010.09.004 21396576

[B65] Constancia M Kelsey G Reik W Resourceful imprinting Nature 2004 432 7013 53 57 10.1038/432053a 15525980

[B66] Silver HK Kiyasu W George J Deamer WC Syndrome of congenital hemihypertrophy, shortness of stature, and elevated urinary gonadotropins Pediatrics 1953 12 4 368 376 13099907

[B67] Russell A A syndrome of intra-uterine dwarfism recognizable at birth with cranio-facial dysostosis, disproportionately short arms, and other anomalies (5 examples) Proc R Soc Med 1954 47 12 1040 1044 13237189

[B68] Abu-Amero S Monk D Frost J Preece M Stanier P Moore GE The genetic aetiology of Silver-Russell syndrome J Med Genet 2008 45 4 193 199 18156438 10.1136/jmg.2007.053017

[B69] Kamp GA Mul D Waelkens JJ Jansen M Delemarre-van de Waal HA Verhoeven-Wind L Frolich M Oostdijk W Wit JM A randomized controlled trial of three years growth hormone and gonadotropin-releasing hormone agonist treatment in children with idiopathic short stature and intrauterine growth retardation J Clin Endocrinol Metab 2001 86 7 2969 2975 10.1210/jc.86.7.2969 11443153

[B70] Hitchins MP Stanier P Preece MA Moore GE Silver-Russell syndrome: a dissection of the genetic aetiology and candidate chromosomal regions J Med Genet 2001 38 12 810 819 10.1136/jmg.38.12.810 11748303 PMC1734774

[B71] Kotzot D Schmitt S Bernasconi F Robinson WP Lurie IW Ilyina H Mehes K Hamel BC Otten BJ Hergersberg M Werder E Schoenle E Schinzel A Uniparental disomy 7 in Silver-Russell syndrome and primordial growth retardation Hum Mol Genet 1995 4 4 583 587 10.1093/hmg/4.4.583 7633407

[B72] Eggermann T Wollmann HA Kuner R Eggermann K Enders H Kaiser P Ranke MB Molecular studies in 37 Silver-Russell syndrome patients: frequency and etiology of uniparental disomy Hum Genet 1997 100 3–4 415 419 9272165 10.1007/s004390050526

[B73] Preece MA Price SM Davies V Clough L Stanier P Trembath RC Moore GE Maternal uniparental disomy 7 in Silver-Russell syndrome J Med Genet 1997 34 1 6 9 10.1136/jmg.34.1.6 9032641 PMC1050838

[B74] Gicquel C Rossignol S Cabrol S Houang M Steunou V Barbu V Danton F Thibaud N Le Merrer M Burglen L Bertrand AM Netchine I Le Bouc Y Epimutation of the telomeric imprinting center region on chromosome 11p15 in Silver-Russell syndrome Nat Genet 2005 37 9 1003 1007 10.1038/ng1629 16086014

[B75] Netchine I Rossignol S Dufourg MN Azzi S Rousseau A Perin L Houang M Steunou V Esteva B Thibaud N Demay MC Danton F Petriczko E Bertrand AM Heinrichs C Carel JC Loeuille GA Pinto G Jacquemont ML Gicquel C Cabrol S Le BY 11p15 imprinting center region 1 loss of methylation is a common and specific cause of typical Russell-Silver syndrome: clinical scoring system and epigenetic-phenotypic correlations J Clin Endocrinol Metab 2007 92 8 3148 3154 10.1210/jc.2007-0354 17504900

[B76] Schonherr N Meyer E Roos A Schmidt A Wollmann HA Eggermann T The centromeric 11p15 imprinting centre is also involved in Silver-Russell syndrome J Med Genet 2007 44 1 59 63 16963484 10.1136/jmg.2006.044370 PMC2597902

[B77] Penaherrera MS Weindler S Van Allen MI Yong SL Metzger DL McGillivray B Boerkoel C Langlois S Robinson WP Methylation profiling in individuals with Russell-Silver syndrome Am J Med Genet A 2010 152A 2 347 355 10.1002/ajmg.a.33204 20082469

[B78] Eggermann T Russell-Silver syndrome Am J Med Genet C Semin Med Genet 2010 154C 3 355 364 10.1002/ajmg.c.30274 20803658

[B79] Georgiades P Chierakul C Ferguson-Smith AC Parental origin effects in human trisomy for chromosome 14q: implications for genomic imprinting J Med Genet 1998 35 10 821 824 10.1136/jmg.35.10.821 9783704 PMC1051456

[B80] Kotzot D Maternal uniparental disomy 14 dissection of the phenotype with respect to rare autosomal recessively inherited traits, trisomy mosaicism, and genomic imprinting Ann Genet 2004 47 3 251 260 10.1016/j.anngen.2004.03.006 15337470

[B81] Simon D Leger J Carel JC Optimal use of growth hormone therapy for maximizing adult height in children born small for gestational age Best Pract Res Clin Endocrinol Metab 2008 22 3 525 537 10.1016/j.beem.2008.03.003 18538291

[B82] de Zegher F Albertsson-Wikland K Wollmann HA Chatelain P Chaussain JL Lofstrom A Jonsson B Rosenfeld RG Growth hormone treatment of short children born small for gestational age: growth responses with continuous and discontinuous regimens over 6 years J Clin Endocrinol Metab 2000 85 8 2816 2821 10.1210/jc.85.8.2816 10946888

[B83] Czernichow P Treatment with growth hormone in short children born with intrauterine growth retardation Endocrine 2001 15 1 39 42 10.1385/ENDO:15:1:039 11572323

[B84] Argente J Gracia R Ibanez L Oliver A Borrajo E Vela A Lopez-Siguero JP Moreno ML Rodriguez-Hierro F Improvement in growth after two years of growth hormone therapy in very young children born small for gestational age and without spontaneous catch-up growth: results of a multicenter, controlled, randomized, open clinical trial J Clin Endocrinol Metab 2007 92 8 3095 3101 10.1210/jc.2007-0078 17536000

[B85] Dahlgren J Wikland KA Final height in short children born small for gestational age treated with growth hormone Pediatr Res 2005 57 2 216 222 10.1203/01.PDR.0000148716.71231.81 15585685

[B86] Carel JC Chatelain P Rochiccioli P Chaussain JL Improvement in adult height after growth hormone treatment in adolescents with short stature born small for gestational age: results of a randomized controlled study J Clin Endocrinol Metab 2003 88 4 1587 1593 10.1210/jc.2002-021123 12679443

[B87] Ranke MB Lindberg A Cowell CT Wikland KA Reiter EO Wilton P Price DA Prediction of response to growth hormone treatment in short children born small for gestational age: analysis of data from KIGS (Pharmacia International Growth Database) J Clin Endocrinol Metab 2003 88 1 125 131 10.1210/jc.2002-020867 12519840

[B88] US Food and Drug Administration: FDA Drug Safety Communication: Ongoing safety review of recombinant human growth hormone (somatropin) and possible increased risk of death. Available at http://www.fda.gov/Drugs/DrugSafety/ucm237773.htm#ds Accessed February 15, 2012

[B89] Sperling MA Long-Term Therapy with Growth Hormone: Bringing Sagacity to SAGHE J Clin Endocrinol Metab 2012 97 1 81 83 10.1210/jc.2011-3271 22223769

[B90] Rosenfeld RG Cohen P Robison LL Bercu BB Clayton P Hoffman AR Radovick S Saenger P Savage MO Wit JM Long-term surveillance of growth hormone therapy J Clin Endocrinol Metab 2012 97 1 68 72 10.1210/jc.2011-2294 22174422

[B91] Wilton P Mattsson AF Darendeliler F Growth hormone treatment in children is not associated with an increase in the incidence of cancer: experience from KIGS (Pfizer International Growth Database) J Pediatr 2010 157 2 265 270 10.1016/j.jpeds.2010.02.028 20400105

[B92] Bell J Parker KL Swinford RD Hoffman AR Maneatis T Lippe B Long-term safety of recombinant human growth hormone in children J Clin Endocrinol Metab 2010 95 1 167 177 10.1210/jc.2009-0178 19906787

[B93] Luger A Feldt-Rasmussen U Abs R Gaillard RC Buchfelder M Trainer P Brue T Lessons Learned from 15 Years of KIMS and 5 Years of ACROSTUDY Hormone Res Paediatrics 2011 76 suppl 1 33 38 10.1159/000329156 21778746

[B94] Loftus J Heatley R Walsh C Dimitri P Systematic review of the clinical effectiveness of genotraopin (somatropin) in children with short stature J Pediatr Endocrinol Metab 2010 23 6 535 551 20662327 10.1515/jpem.2010.092

[B95] Ergun-Longmire B Mertens AC Mitby P Qin J Heller G Shi W Yasui Y Robison LL Sklar CA Growth hormone treatment and risk of second neoplasms in the childhood cancer survivor J Clin Endocrinol Metab 2006 91 9 3494 3498 10.1210/jc.2006-0656 16822820

